# Verdict of Court-Martial

**Published:** 1885-09

**Authors:** 


VERDICT OF COURT-MARTIAL.

The verdict of the court-martial in the case of ex-Surgeon General Wales has, says a Washington correspondent of the Philadelphia *Medical Times*, caused somewhat of a sensation. It will be remembered that Dr. Wales was tried by court-martial and found guilty of inefficiency and neglect of duty, because he failed to discover the rascality of several of his clerks, by which the Government was swindled out of a large amount of money, said to be over a hundred thousand dollars. The court failed to connect Dr. Wales with any of the frauds, but convicted him on a technicality, and imposed what is considered here a very severe penalty, out of all proportion to the offence. The high professional character of the late Surgeon General, and his successful labors for the advancement of the medical staff of the navy, did not avail him; in fact, they seemed to render his offence the greater in the eyes of the court, which apparently held the opinion that the duties of the Surgeon General were simply to watch his subordinates and prevent their stealing. It seems by the confession of the principal criminal, the late chief clerk of the Bureau of Medicine and Surgery, that the frauds had gone on undetected for years under Dr. Wales's predecessors; but the court refused to go into the inquiry farther than concerned the latter's administration. To outsiders it looks very much as if Dr. Wales was the victim of a systematic persecution, of which the late Secretary of the Navy was the chief promoter. It is well known here that there was a violent personal antipathy between the two men, which may have been partly political, but it is believed to have largely arisen from social antagonisms. The penalty imposed by the court is suspension from rank and duty for a period of five years. It is said that Dr. Wales's counsel will not rest satisfied with this verdict, but will appeal the case to the Supreme Court.—*New York Medical Record.*

